# Conserved catalytic motifs encode enzyme-like supramolecular peptide assemblies

**DOI:** 10.21203/rs.3.rs-10744902/v1

**Published:** 2026-08-25

**Authors:** Liam R. Marshall, Sagar Bhattacharya, Leonardo F. Serafim, Parth Rathee, Malitha C. Dickwella Widanage, Prerana Dash, Yang Li, Ho Yee Joyce Fung, Souvik Panda Mahapatra, Marianne L. Jensen, Lars Hemmingsen, Tuo Wang, Rajeev Prabhakar, Ivan V. Korendovych

**Affiliations:** 1 Department of Chemistry and Biochemistry; Baylor University, Waco, TX, USA; 2 Department of Chemistry, University of Miami; Coral Gable, FL, USA; 3 Department of Chemistry, Michigan State University; East Lansing, MI, USA; 4 Department of Biophysics, University of Texas Southwestern Medical Center; Dallas, TX, USA; 5 Department of Chemistry, University of Copenhagen; Copenhagen, Denmark

## Abstract

A seven-residue fragment derived from the active-site region of carbonic anhydrase spontaneously forms Zn^2+^-binding amyloid fibrils that catalyze carbon dioxide hydration with catalytic efficiencies surpassing all previous carbonic anhydrase mimics and approaching those of natural enzymes. Cryo-electron microscopy at 2.2 Å resolution, supported by perturbed angular correlation spectroscopy, solid-state NMR, molecular dynamics simulations and QM/MM calculations, reveals a supramolecular active site that recapitulates key structural and mechanistic features of the enzyme despite arising from a fundamentally different protein fold. Guided by structure–activity relationships, minimal sequence modifications fallowed for further optimization to reach carbon dioxide hydration activity of 1.3 × 10^6^ M^−1^ s^−1^. Extending this strategy to a conserved motif from superoxide dismutase yielded copper-binding catalytic amyloids that promote superoxide dismutation near the diffusion limit. Together, these findings establish conserved catalytic motifs as a rich source of functional catalysts and demonstrate that they encode sufficient information to construct highly active supramolecular active sites independently of the globular protein fold. More broadly, they demonstrate that complex catalytic function can emerge from remarkably simple self-assembling peptide architectures, providing an experimentally tractable framework for investigating the fundamental principles that underlie enzymatic catalysis.

Modern enzymes achieve extraordinary catalysis through precisely organized active sites within complex protein folds, but early functional peptides likely lacked such structural sophistication.^1,2^ This raises the question of how efficient catalysis emerged before folded proteins evolved, and whether self-assembly provided an accessible route to organized, functional structures.^3-5^ Short β-sheet peptide assemblies provide an attractive system for addressing this question because very short peptides spontaneously assemble into diverse, ordered supramolecular architectures, including catalytic amyloids.^6-11^ The recent discovery of high catalytic activities in model reactions exerted by amyloid-like assemblies further supports the connection between peptide assemblies and early enzymes.^8,10,12-14^ However, it remains unclear whether catalytic amyloids can recapitulate the structural and mechanistic features that underlie the extraordinary catalytic efficiencies of modern enzymes.

Carbonic anhydrases that facilitate the equilibrium between carbon dioxide and water are absolutely critical for carbon-based life and therefore are present in all known organisms, with a wealth of functional and structural information available for different classes of these enzymes that are presumed to have evolved in a convergent fashion.^15^ CA’s rely on a concerted action of a single metal ion and an intricate arrangement of amino acid side chains to increase the nucleophilicity of metal bound water and to promote CO_2_ hydration.^16^ The dominant structural classes vary between the different kingdoms of life, but the metal binding motif of the α-class of CAs, that are found in all organisms, has a consensus sequence QFHFH remarkably conserved across all kingdoms of life (Fig. 1, Fig. S1, Supplementary Information). The consensus sequence is even longer when CA‘s of different kingdoms are considered separately, e.g. QFHFHWG is found in the most characterized *Animalia* enzymes (Fig. 1). This fragment shows a classic alternating hydrophilic/hydrophobic residue pattern, prompting us to ask whether these conserved catalytic motifs retain sufficient information to direct the assembly of functional supramolecular active sites outside the context of the native protein fold.

Synthetically prepared Ac-QFHFHWG-NH_2_ (**1**) self-assembles at near neutral pH to form well defined β-sheet rich fibrils as demonstrated by transmission electron microscopy (TEM), circular dichroism (CD) and thioflavin T (ThT) fluorescence data (Fig. 1, Fig. S2-S6, Supplementary Information). Moreover, when the peptide self-assembles in the presence of Zn^2+^, the resulting fibrils promote ester hydrolysis (Table 1, Fig. S3a, Supplementary Information). Encouraged by this finding we tested the assemblies formed by **1** in the presence of Zn^2+^ for carbon dioxide hydration. The dependence of the initial rate of reaction on carbon dioxide concentration follows the Michaelis-Menten model (Fig. 1c) with the catalytic efficiency (k_cat_/K_M_) of 3.3 ± 1.2 x 10^5^ M^−1^s^−1^. This level of activity is quite remarkable – it is to our knowledge the highest reported to date in any CA mimic.^8,16-18^ In fact, considering the low molecular weight of the active unit, **1-**Zn^2+^ has a specific activity that is right in the middle of the range exhibited by naturally occurring enzymes (Table 1). The observed catalytic activity is solely due to the self-assembled species: after centrifugation at 100,000 g the supernatant showed no catalytic activity (Fig. S7, Supplementary Information).

Next, we have undertaken a structure-morphology-activity study to better understand the different factors that influence catalysis. Preserving the His/His/Gln motif while changing the hydrophobic core to phenylalanine only (peptides **2** and **3**) resulted in increased CO_2_ hydration activity, especially when the Gln was positioned on the C-terminal side of the metal binding motif. Previously we and others observed that K_M_ values for hydrolytic reactions promoted by related self-assembling peptides can be lowered by incorporation of tyrosine residues.^19,20^ Moreover, tyrosine plays a prominent role for proton transfer in native carbonic anhydrases.^21^ Substituting glutamine for tyrosine had a sequence dependent impact on catalytic parameters – in position two (peptide **4**) it lowered K_M_, yet in position six (peptide **5**) it led to significant drop in CO_2_ hydration activity, albeit with drastically increased ability to promote ester hydrolysis. Next, we explored how these limited sequence activity relationships can be prospectively applied to improve the activity further by extending the sequence of the HFHF zinc binding core by adding FY at the N-terminus and QF at the C-terminus to simultaneously increase the k_cat_ and lower the K_M_. The resulting 9-residue peptide **6** (Ac-FYFHFHFQF-NH_2_) showed catalytic efficiency in CO_2_ hydration of 1.29 ± 0.39 x 10^6^ M^−1^s^−1^, that is nearly quadruple that of **1**-Zn^2+^, exceeds the reported value of HCAIII and is on par with HCAII in terms of specific activity. Interestingly, the K_M_ value for CO_2_ hydration shown by the most active assemblies is very similar to the one found in natural enzymes. Like all previously reported functional model of carbonic anhydrases, the effective pK_a_ of the catalytic nucleophile is fairly high- ~ 8.7 as determined from the pH profile of its hydrolytic activity (Fig. 2H).^17,18^ Despite high sequence similarity, the fibrils formed by different peptides have distinct morphologies as shown by TEM and CD. Supramolecular assembly is key to catalysis: introduction of methyl groups into the backbone amides to preclude b-sheet formation produces hydrolytically inactive peptide Ac-FYF_NMe_HF_NMe_HFQF-NH_2_ (Fig. S11, Supplementary Information). The subtle backbone and side chain modification are clearly important for activity as closely structurally related assemblies formed by Ac-IHIHQI-NH_2_ (as well as all others tested) show no CA activity, despite having a remarkably similar metal coordination sphere to the one seen in carbonic anhydrase that served as an inspiration for its design (Fig 1a).^11,22,23^ Cryo-EM analysis of **1** produced a 2.2 Å resolution map of the supramolecular assemblies formed by **1**-Zn^2+^ (Fig. 1i-l, Table S2, Fig. S8, Supplementary Information). It shows a well-defined double helical structure formed by a tetrameric peptidic unit. The strands are arranged in a very rare parallel/parallel form (Class 3) with the N-terminal glutamine residues tightly packing against each other forming a ternary fold. The electron density is best modelled by averaging two different conformations of metal coordinating histidines (Fig. 2). The metal binding site could not be unambiguously modelled from cryo-EM data alone but is consistent with a tetrahedral zinc binding site with a nucleophilic water molecule bound to the zinc ion (Fig. 1) supported by ^111m^Cd PAC spectroscopy (Table S3, Fig. S9, Supplementary Information). The PAC signal is described by a single low frequency nuclear quadrupole interaction (NQI) with the parameters *ω*_0_ = 0.069 rad/ns and *η* = 0.30, that are in excellent agreement with the predicted values for the model (*ω*_0_ = 0.075 rad/ns and *η* = 0.47). The apparent inequivalence of the His side chains in the two types of peptide strands pronounced in the cryo-EM structure is a necessary result of the requirement for the tetrahedral metal ions site that require symmetry breaking when using a two-His monomeric unit. This is commonly observed in homodimeric enzymes in nature, where the two monomers have nearly identical structures, except near the active site, where symmetry is necessarily lifted to create a catalytic site (e.g., in HIV protease).

To gain insight into molecular determinants of the enzymatic level catalysis showed by the self-assembling peptides we performed structural and computational studies on the most catalytically active fibrils formed by **6**. The chemical shifts of the backbone atoms obtained from solid state NMR (ssNMR) experiments show perfect agreement with a typical cross β-sheet structure and provided us with backbone dihedral angles for subsequent structural modeling. We have also observed two different sets of histidine ^15^N chemical shifts, one indicative of a bridging histidine residue, consistent with the two types of zinc sites observed previously in a ssNMR structure of a related catalytic amyloid (Ac-IHVHLQI-NH_2_).^22^ The ssNMR-derived constraints were then used to build a model of **6**-Zn^2+^ using molecular dynamics (MD) simulations (Fig. 3d). The 12-peptide unit assembly was modeled as a bilayer and equilibrated using 500 ns all-atom MD simulations (Fig. 2b). The resulting structure adopted a twisted (twist angle of ~6.5°) cross - β fibril supported by hydrogen bonds between the adjacent monomers (Fig. 2b) stabilized by interstrand hydrogen bonds and a tight hydrophobic core formed by intercalating, zipper-like phenylalanine residues. The Zn^2+^ ions coordinate to three His residues, just like in CA, however unlike the enzyme, the zinc sites are connected by bridging, deprotonated histidine, that lowers the overall charge and contributes to peptide assembly stability (Fig. 2e). The vacant coordination sites are occupied by water molecules. The non-bridging His residue forms a hydrogen bond with the side chain of Gln.

While structural studies unequivocally point to two types of zinc ions in the fibrils with 1:1 metal to peptide stoichiometry, the dependence of activity on the zinc concentration is consistent with 1:2 metal to peptide stoichiometry (Fig. S10, Supplementary Information). This discrepancy is consistent with different functional properties of the two metal sites: one site being catalytic (C-site) and the other one playing a purely structural role (S-site). Analysis of the MD model is very much in agreement with this hypothesis. In the C-site, a Tyr residue is ideally positioned to polarize the Zn^2+^-bound water molecule and generate the hydroxyl nucleophile (**R** state, Fig. 2f). The metal binding and interaction with the Tyr residue substantially lowers the pK_a_ value of the water and facilitates spontaneous formation of the nucleophile, a general strategy employed by many hydrolytic enzymes.^25^ To further probe mechanistic aspects of catalysis promoted by the peptide assembly we performed quantum mechanics/molecular mechanics (QM/MM) calculations of CO_2_ hydration. In the first step, the hydroxyl nucleophile attacks a CO_2_ molecule to generate a Zn^2+^-bound bicarbonate species containing intermediate (**I**) with a barrier of 8.8 kcal/mol (Fig. 2g). In **I**, the hydroxyl nucleophile doesn’t leave the metal ion, but the Zn-O distance is significantly elongated by 0.09 Å. Additionally, it is stabilized by hydrogen bonding with the surrounding water molecules that mimics the role of the second coordination shell Thr199 in carbonic anhydrase. This step is endergonic by 5.3 kcal/mol due to the unfavorable positioning of the hydroxyl proton in **I**. In the next step, this proton can be transferred through either Lipscomb (via bridging water) or Lindskog (through the rotation of the bicarbonate group) mechanisms.^26-28^ The barrier for the Lipscomb mechanism is computed to be 5.0 kcal/mol lower than the one for the Lindskog mechanism. From **I**, the proton transfer via transition state (**T2**) occurs with a barrier of 7.7 kcal/mol *i.e.* 13.0 kcal/mol from **R**. It is the rate-determining step of the entire mechanism as proposed for the mechanism of CA.^29^ Additionally, the computed barrier is only slightly higher than the barrier computed for the enzyme.^29^ The creation of the bicarbonate ion in the product (**P**) is thermoneutral. In **P**, the bicarbonate is coordinated to the Zn^2+^ and stabilized by water molecules from the adjacent non-catalytic sites followed by its replacement by water to initiate the next cycle. To establish whether highly active catalytic amyloids can be derived from sequences of other natural proteins, we looked at another ancient, essential and highly conserved family of enzymes – superoxide dismutase (Fig. S14, Supplementary Information). These enzymes incorporate a redox active metal ion (usually copper) in their active site and are amongst the fastest enzymes known, with typical catalytic efficiencies above 10^9^ M^−1^ s^−1^. Copper ions promote this reaction efficiently but sequestering it within an enzyme lowers cellular toxicity and tunes the properties of the metal site to achieve maximum efficiency. Analysis of the sequences of archaeal superoxide dismutases reveals a highly concerted HGIHIH motif (Fig. 3). Synthetically prepared peptide Ac-HGIHIH-NH_2_ (**7**) self-assembles in the presence of copper as indicated by CD, ThT and electron microscopy data (Fig 3, Fig S15, Supplementary Information). The assemblies formed by **7** in the presence of copper are highly reactive in promoting superoxide dismutation, approaching the diffusion limit, with the kMcF of 1.4 x 10^8^ M^−1^s^−1^. SOD activity in various model systems has been extensively benchmarked and this level of activity is the highest among all SOD peptide mimics under the same conditions (Table S4). The observed superoxide dismutase activity of **7** is due solely to the self-assembled species, as the supernatant obtained after centrifugation at 100,000 g shows no activity above the blank (Fig. S16, Supplementary Information). Non-self-assembling peptides H_2_N-HGIHIH-NH_2_ (**7a**) and Ac-HAAHVH-NH_2_ (**8**) with the same pattern of metal coordinating residues show much diminished activity (Figure 3D, Figures S17, S18 Supplementary Information) further supporting a correlation between activity and self-assembly.

The high catalytic activity of amyloids formed by peptides derived from natural proteins begs the question whether a possible evolutionary connection between short amyloids and modern-day proteins could manifest itself in amyloids formed by natural proteins. Amyloids formed by SOD1 have been previously characterized structurally, so we tested them for superoxide dismutase activity. The reported cryoEM structure of full-length SOD1 fibrils shows the conserved active site present in a disordered region of the amyloid fibril, accessible to the substrate.^30^ Upon fibrillization, fibrils formed by SOD1 promote superoxide dismutation with k_McF_ comparable to that shown by **7** possibly indicating similarity of the structures observed in the fibrils and the peptide assemblies (Fig 3B). While enzymes aggregated though domain swapping and the introduction of tags have been shown to retain their catalytic activity, demonstrating this with a native enzyme in an amyloid state has not been to our knowledge reported before.^31^ This observation contributes to the ongoing discussion of the role of amyloids in the Anfinsen’s thermodynamic picture.^32-35^

Despite the explosive growth of structural and functional knowledge on proteins, mere replication of enzymatic activity in simplified systems remains elusive and the solution to the problem of creating efficient enzymes *a la carte* is still not available.^36-39^ Thus, so far *all* major successes in the enzyme design field have been using unnatural, and in the vast majority of cases activated, substrates.^40-42^ Here we demonstrate that conserved catalytic motifs derived from natural enzymes retain sufficient information to reconstruct highly active supramolecular catalysts outside the globular protein fold. Self-assembly is key as mere replication of the sequence is not sufficient to achieving high activity. Amyloid-like peptide assemblies serve as a functional type of protein fold that supports productive arrangements of functional groups, including water molecules, akin to what is found in natural enzymes. Even in such simple systems, minor modifications of peptide sequences allow for accessing various assembly morphologies with various levels of activities. Despite differences in the metal coordination between catalytic amyloids and natural enzymes, the overall mechanism appears to be quite similar as indicated by QM/MM studies, supporting the notion that prebiotic peptide assemblies could effectively promote chemical transformations. These findings establish conserved enzyme motifs as a rich source of functional catalytic assemblies and provide a general strategy for designing efficient supramolecular enzymes. The ease with which we discovered highly efficient supramolecular catalysts of important chemical transformations opens possibilities for rapid discovery of an array of practically applicable peptide catalysts. More broadly, they demonstrate that complex catalytic function can emerge from remarkably simple self-assembling peptide architectures, providing an experimentally tractable framework for investigating the fundamental principles that underlie enzymatic catalysis.

## Methods

### Chemicals and reagents

All reagents and buffers were purchased from Ambeed, Fisher Scientific, VWR, Sigma Aldrich, Chem-Impex, Biobasic, Inc. and Santa Cruz Biotechnology, Inc. and used without additional purification. Buffers were made using MilliQ water (Millipore Elix 3 instrument). Superoxide dismutase and xanthine oxidase (from bovines) were obtained from Sigma Aldrich. Labeled ^15^N and ^13^C amino acids were purchased from CortecNet.

### Multiple Sequence Alignment (MSA) analysis

We used carbonic anhydrase from *T. ammonificans* (PDB 4COQ), copper superoxide dismutase (CuSOD) from *C. albicans* (PDB 4N3U) or copper-zinc superoxide dismutase (Cu/Zn SOD) from *H. sapiens* (PDB 4B3E) as starting points for structural searches. Protein homologs were identified using the NCBI BLASTP webserver with standard search parameters, except increasing maximum results to 5000. (https://blast.ncbi.nlm.nih.gov/Blast.cgi) Results were partitioned according to taxonomic kingdom. Protist sequences were specifically analyzed using the Chinese National Center for Bioinformation P10K BLAST. (https://ngdc.cncb.ac.cn/blast/p10k/). We removed duplicate sequences, and aligned the data using MAFFT (v7, https://mafft.cbrc.jp/alignment/server/large.html).^43^ Sequence conservation profiles were rendered using a custom Python visualization tool. Protein sequence alignments generated from BLAST-derived datasets were imported into the software in FASTA format and processed to remove duplicate sequences and non-informative alignment positions. The alignment tool parsed each column of the multiple sequence alignment (MSA) and calculated amino acid frequencies at each position. Sequence logos were generated from the resulting residue frequency matrices using an information-content-based representation, in which the height of each amino acid symbol reflects its relative conservation within the alignment. For each alignment position, amino acid frequencies were converted to information content values using Shannon entropy calculations:

$$R_{i}=\log_{2}(K)-H_{i}$$

where (R_i_) represents the total information content at position *i*, and (H_i_) is the Shannon entropy of the amino acid distribution at that position. Individual residue heights were calculated by multiplying the information content by the fractional abundance of each amino acid at that position. Thus, highly conserved residues produced larger symbols, whereas variable positions produced smaller or absent symbols. The resulting sequence logos were rendered directly within the custom alignment visualization tool using the calculated residue matrices. Logos were formatted with consistent dimensions, residue coloring, and axis parameters to enable comparison between independent sequence datasets. Alignment regions were exported as high-resolution vector graphics for subsequent figure preparation.

### Peptide synthesis and characterization.

Peptides were synthesized using fluorenylmethyloxycarbonyl (Fmoc) solid-phase synthesis, as previously described for the synthesis of hydrophobic peptides.^44^ Rink amide resin (0.65 mmol/g) was swollen for 20 min before synthesis. Each synthetic step consisted of: 1) Double Fmoc deprotection using 20 % 4-methyl piperidine in DMF 2) coupling of target amino acid using a mixture of DIPEA and HCTU for 20 min 3) washing twice in DMF 4) N-terminal acetylation for 5 min with a capping solution (DIPEA: acetic anhydride: DMF; 2:1:100) to react with any unreacted N-termini. After synthesis, the resin was washed with dichloromethane and dried under a stream of nitrogen. Resin cleavage and sidechain deprotection was achieved simultaneously by treatment with a mixture of trifluoroacetic acid (TFA), triisopropylsilane (TIPS) and water (95:2.5:2.5 v/v) for 2 h at room temperature. The crude peptides were precipitated and washed with ice cold diethyl ether and purified using a reverse phase high-performance liquid chromatography (HPLC) system (Varian ProStar 210) with a C4 preparative column (Vydac) using a linear gradient of elution solvent A (0.1 % TFA, 99.9% Millipore H_2_O) and solvent B (90% acetonitrile, 9.9% H_2_O, 0.1 % TFA). Identities were confirmed using matrix-assisted laser desorption ionization time-of-flight (MALDI-TOF) mass spectrometry on a Bruker Autoflex III Smartbeam MALDI-TOF spectrometer. Purity was evaluated with an Agilent 1260 instrument with an analytical Zorbax Eclipse XDB-C18 column (2.6 mm x 150 mm).

### Preparation of peptide stocks and solutions.

Purified lyophilized peptides were dissolved in 10 mM hydrochloric acid to make a 1 mM stock solution, with concentrations determined using the absorbance at 214 nm (and 280 nm for peptides containing Trp) on an Agilent Cary 60 UV-Vis spectrophotometer. Extinction coefficients for the peptides at these wavelengths were calculated using literature values.^45^ The buffered working stocks of the peptides were prepared by dilution of the stock peptide solution into the appropriate buffer. Working solutions were prepared immediately before use in the case of 0 h experiments, or prepared and then aged on the bench at room temperature.

### SOD amyloid formation and purification.

Full-length bovine SOD1 was fibrillated using a method adapted from Wang *et. al.*^30^(*27*) In short, full length bovine Cu-Zn SOD (30 μM) (Sigma Aldrich) was incubated in 20 mM Tris-HCl buffer (pH 7.4) with 5 mM TCEP and shaken at 37 °C for 48 hours. These fibril solutions were then diluted to 10 mL and dialyzed in cassettes (100 kDa MWCO) against 4L of fresh buffer (50 mM HEPES, pH 7.8) for 24 hours (3 x buffer changes) to exclude any remaining folded enzyme. The samples were then concentrated to ~2 mM in a centrifugal filter (VWR). The concentration of the fibrils was verified using the extinction coefficient at 280 nm (1490 M^−1^ cm^−1^) using an Agilent Cary 60 UV-Vis spectrophotometer before use in assays.

### pNPA hydrolysis.

Kinetic measurements were performed using a BioTek Eon microplate spectrophotometer by monitoring the increase in absorbance from the product (*p*-nitrophenol) at 405 nm at 25 °C in 96-well plates. *p*-nitrophenol acetate was used from a 0.1 M stock in acetonitrile, with final acetonitrile concentration 2% in all reaction mixtures. 50 μL of the working peptide solution was pipetted into wells, with 150 μL of freshly prepared substrate solution (0.195-0.75 mM) added to initiate the reaction. The reported results were obtained by averaging three independent measurements of two peptide stocks. Extinction coefficients of 1,700, 4,300, 9,100, 12,700 and 16,600 M^−1^cm^−1^ were used to determine the product concentration at pH values of 6, 6.5, 7, 7.5 and 8, respectively, at 405 nm.^11^(*11*) An extinction coefficient of 18,700 M^−1^cm^−1^ was used at pH 8.5 and above at 405 nm. Kinetic parameters (*k*_cat_ and *K*_M_) were obtained by fitting the data to the Michaelis–Menten equation (*v*_0_ = *k*_cat_[*E*]_0_[*S*]_0_/(*K*_M_ + [*S*_0_])). pH profile studies were performed using the following buffers at a concentration of 25 mM: MES (pH 6–6.5), HEPES (pH 7–7.5), Tris (pH 8–8.5), TAPS (pH 9–9.5) and CAPS (pH 10–10.5)) Product formation was monitored for 5 min every 30 s. The pH profile data were fitted the following equation:

$$\frac{k_{cat}}{K_{M}}=\frac{{\left(k_{cat}∕K_{M}\right)}_{max}*10^{-pKa}}{10^{-pH}+10^{-pKa}}$$

where *k*_cat_/*K*_M_ is the observed enzymatic efficiency at a particular pH value, p*K*_a_ is the p*K*_a_ value for the protonation/deprotonation step and (*k*_cat_/*K*_M_)_max_ is the corresponding maximum activity.

### CO_2_ hydration kinetics.

CO_2_ hydration was performed following the matched indicator procedure first described by Khalifah.^17,46^ Buffer-indicator pairs used were as follows: pH 9.5 CHES/thymol blue (590 nm). Each sample was prepared in 0.1 M buffer, 50 μM indicator, and 5 μM peptide-Zn complex, to yield in cell concentrations of 50 mM buffer, 25 μM indicator, and 2.5 μM Zn-peptide complex. We followed the buffer factor determination described by Pecoraro and co-workers and found our buffer factor in agreement with their findings (0.09).^17,18^ CO_2_ solutions were prepared by bubbling through MilliQ water for ten minutes with the use of a fritted glass gas bubbler. For kinetic characterization, a stock of saturated CO_2_ (33 mM) was prepared freshly each time and then diluted to the required concentration. Dilutions were carried out by mixing MilliQ water with the saturated CO_2_ in gas tight syringes. Experiments were performed by mixing peptide/metal/buffer/indicator or buffer/peptide/indicator (in the case of blanks) solutions in equal proportions with aqueous CO_2_ solutions. Upon mixing, 10 seconds of absorbance data were recorded, but only the initial linear portion of the curve was used to calculate initial rates. After calculating the slope of the initial portion, these were multiplied by our calculated buffer factor to yield the initial rate. At least 5 repeats for each concentration were recorded and averaged, and blank shots for each concentration are subtracted from the peptide shots in the calculation of the Michaelis-Menten parameters.

### Superoxide dismutase activity

The superoxide dismutase (SOD)-like function of the tested peptides was evaluated by their ability to inhibit the reduction of nitro blue tetrazolium (NBT) by superoxide radicals, which were enzymatically generated in situ using the xanthine/xanthine oxidase system.^47^ In this assay, superoxide radicals produced from the oxidation of xanthine by xanthine oxidase reduce NBT to a blue-colored formazan product. This reaction is monitored by measuring the increase in absorbance at 560 nm (Agilent BioTek Synergy H1 plate-reader). All experiments were performed in 50 mM HEPES buffer at pH 7.8, containing a final concentration of 250 μM NBT and 37.5 μM xanthine. The reaction was initiated by adding 50 μL of xanthine oxidase (XO) solution—comprised of 4 925 μL of 50 mM HEPES buffer (pH 8) and 75 μL of XO—to 50 μL of Solution 2, which contained 5 mL of 50 mM HEPES (pH 8), 5 mL of 2 mM NBT (in the same buffer), and 75 μL of xanthine from a 200 μM stock. Under these control conditions (without enzyme or copper), this mixture consistently produced a reduction in NBT at a rate of 4 × 10^−4^ OD_560_ per second. To assess superoxide scavenging activity, peptide samples complexed with Cu(II) were tested across a concentration series from 100 μM down to 0.01 nM. Kinetic measurements of absorbance at 560 nm were recorded continuously over a three-minute interval, and the initial rate of change in OD_560_ was used to quantify the extent to which each peptide–copper complex inhibited NBT reduction relative to the control.

SOD like activity was quantified by determining the IC_50_ value, which represents the concentration required to achieve 50% suppression of NBT reduction. IC_50_ values were determined by plotting percentage of inhibition against the concentration of peptide-Cu(II) complex analyzing the resulting curve by dose dependent Hill plot. The data were fit to the following equation using GraphPad:

$$Y=A_{\text{min}}+\left(A_{\max}\;\text{-}\;A_{\text{min}}\right)∕(1+10^{\left({\left({\mathrm{logIC}}_{50}\;\text{-}\;X\right)}^{*}\text{Hill Slope}\right)})$$

where, Y: Observed response, X: Logarithm (base 10) of the peptide-Cu(II) complex concentration, A_max_: Maximum response plateau, A_min_: Minimum response plateau, LogIC_50_: Logarithm of the inhibitor concentration that produces a response halfway between Top and Bottom, Hill Slope: Slope factor indicating the steepness of the curve.

### Ultracentrifugation.

Samples were prepared as described above. Samples were centrifuged at 100,000 g for 1 hour (Beckman Optima XPN1000**).** After ultracentrifugation, the supernatant was decanted from the centrifuge tubes and used for subsequent experiments.

### Circular Dichroism.

CD spectra were collected on a Jasco J-715 CD spectrometer using continuous scan mode and a quartz cuvette with a 0.1 cm path length. Each spectrum is an average of four scans. Samples contained peptide (25 μM) and ZnCl_2_ (25 μM) in buffer (5 mM HEPES, pH 8). Care was taken so the sample absorbance never exceeded 1.5 at all wavelengths to produce reliable ellipticity values.

### Thioflavin T fluorescence.

Fluorescence spectra were obtained on an Agilent Cary Eclipse fluorescence spectrophotometer operating in the steady-state mode at 25°C with emission and excitation band width set to 5 nm. The measurements were taken using a quartz cuvette with 5 mm excitation and 5 mm emission path lengths. Samples were prepared by mixing peptide (1 mM stock at pH 2, 20 μL) with buffer (25 mM Tris, pH 8.0, 0.1 mM Zn) to a final concentration of 100 uM, and ThT (2 mM) was then added to a final concentration of 25 μM, and the samples were vortexed before measurement.

### Transmission Electron Microscopy.

Peptide samples were prepared according to the same procedures described in the kinetic assays. Sample aliquots of 10 μl were adsorbed onto glow-discharged carbon-coated, 200-mesh copper grids (Ted Pella) for 2-5 minutes. Grids were then stained with two 5 μl drops of 2% (w/v) uranyl acetate. After drying, samples were viewed with a JEOL-JEM 2100F Field Emission electron microscope at an acceleration voltage of 200 kV. Electron micrographs were recorded on a Gatan OneView 4K CCD camera.

### Solid-state NMR

Solid-state NMR experiments were conducted on a Bruker Avance 400 MHz (9.4 T) spectrometer equipped with a 4 mm MAS HCN probe. Spectra were acquired under magic-angle spinning (MAS) conditions at 11.2 kHz for ^13^C and 10 kHz for ^15^N at 297 K. ^13^C chemical shifts were externally referenced to the TMS scale by calibrating the CH_2_ signal of adamantane to 38.48 ppm. ^15^N chemical shifts were referenced to the liquid ammonia scale by calibrating the methionine amide resonance of N-formyl-Met-Leu-Phe-OH to 127.88 ppm. Typical radiofrequency field strengths were 80-100 kHz for ^1^H decoupling, 62.5 kHz for ^1^H hard pulses, 50-62.5 kHz for ^13^C, and 41 kHz for ^15^N. One-dimensional (1D) spectra were recorded using cross-polarization (CP) with contact times of 1 ms for ^13^C and 1.5 ms for ^15^N. Four different peptides labeled with U-^13^C,^15^N amino acids in positions a) 1 and 4 (denoted F1H4); b) 2 and 6, respectively (denoted Y2H6); c) 3 and 8 (denoted F3Q8); and d) 5 (denoted F5) were used for chemical shift assignments. Spectral deconvolution of ^15^N CP spectra for Phe1/His4 and Tyr2/His6 residues was performed using DMfit software (version dmfit#20191211) to resolve signals from the minor conformers.^48^ Two-dimensional (2D) ^13^C-^13^C PDSD spectra with a 50 ms mixing time were conducted for resonance assignments. Backbone torsion angles (φ and ψ) of the fibrils were predicted using TALOS-N.^49^

### Computational Procedure

The solid-state NMR structure of the IHVHLQI amyloid (PDB ID: 5UGK) was used as the starting point to build a model for the FYFHFHFQF amyloid.^22^ The IHVHLQI peptide was first mutated to generate the FYFHFHFQF sequence and then replicated into a bilayer with 6 strands followed by addition of terminal caps. In the first step, the bilayer in the metal-free form was equilibrated for 200 ns through all-atom MD simulations in an aqueous solution treated using the TIP4PEW water model. ^50^ These simulations were performed utilizing both CHARMM36m and AMBER14sbFF force fields using the GROMACS 2022 program.^51-53^ In the next step, the Zn^2+^ ions were added to the MD equilibrated structure to build assembly of Zn^2+^-HIS_3_ metal centers using the neutral (HID) and charged (HIX) forms of HIS residues on the solvent exposed side of the bilayer (Fig. S12). These metal centers were connected by the charged HIX residues. HIX was parametrized separately for both CHARMM36m and AMBER14sbFF forcefields. The structure of HIX was optimized at the MP2/6-31+G** level of theory in gas-phase using the GAUSSIAN 16 program.^54,55^ The optimized structure was then used to compute Merz-Kollman RESP charges.^56^ For this residue, the CHARMM36 forcefield parameters were computed using the CHARMM General Force Field (CGENFF) algorithm while Antechamber module and Generalized Amber Force Field (GAFF) for AMBER parameters.^57,58^

In the models of **6**-Zn^2+^ amyloid, ψ and φ dihedral constraints were taken from ^13^C and ^15^N NMR experiments and they were again equilibrated for 500 ns under the aforementioned conditions. In MD simulations, the coulombic and Van Der Waals interactions were calculated using the smooth Particle Mesh Ewald method or SPME with a cutoff of 10.0 Å each.^59,60^ Appropriate isotropic Pressure Coupling and Temperature Coupling re-scalable models (Velocity Rescale thermostat and C-Rescale barostats) were utilized for equilibration.^61,62^ All MD simulations were run with a step size of 2 fs/step in a Periodic Boundary Condition (PBC) corrected cubic box of dimension 9 Å. Structural constraints were calculated with the LINCS algorithm.^63^ The computed root-mean-square-deviations (RMSD) confirmed the equilibration of trajectories within the simulation time frames. Important parameters like hydrogen bonding patterns, solvent accessible surface area and backbone dihedrals were used to analyze the results. In these structures, the hydrophobic sidechains of top and bottom sheets intercalate into a zipper like conformation to minimize solvent accessible space. They form on average 95-97 hydrogen bonds to stabilize the overall structure. The Define Secondary Structure of Proteins (DSSP) analysis showed a predominantly β-sheet rich structure (> 90%). The amyloid provides approximately 69.8 nm^2^ of solvent exposed surface area with high contributions from TYR, HID, HIX and GLN residues. The most equilibrated structures provided by these simulations were subsequently used for QM/MM calculations using two-layer hybrid ONIOM method.

All hybrid two-layer QM/MM (ONIOM) calculations were performed using the Gaussian 16 software package.^55,64-66^ The QM region includes both catalytic and non-catalytic metal sites formed by two Zn^2+^ ions, five histidines, one tyrosine and one glutamine residues. Based on their locations in MD simulations, five water molecules surrounding the metal centers are also incorporated in the quantum region. The ONIOM optimization procedure uses macro/micro interactions, and the electrostatic interactions between the QM and the MM parts were treated using electronic embedding.^67^ The QM region was optimized without any geometric constraint using the hybrid B3LYP exchange-correlation functional.^68^ The Lanl2Dz basis set that includes the Hay-Wadt effective core potential (ECP) was used for the Zn atoms and the 6-31G basis for all other atoms.^69,70^ Grimme’s function with the Becke-Johnson damping effect (GD3BJ) was utilized to incorporate the dispersion effects.^71^ The remaining atoms were included in the MM region and modeled using the AMBER forcefield as implemented in Gaussian 16 software. Hessians were calculated at the same level of theory as the optimizations to confirm the nature of the stationary points along the reaction coordinate. The transition states were confirmed to have only one imaginary frequency corresponding to the reaction coordinates. Zero-point vibrational, thermal, and entropy corrections were added to the final energies (at 298.15 K and 1 atm).

### Cryo-EM sample preparation

Ac-QFHFHWG-NH_2_ (1.2 mM peptide, 1.2 mM Zn, 25 mM Tris, pH 8) was prepared as described prior. The protein sample was then applied to Quantifoil 300 Copper mesh R1.2/1.3 grids (SPI) pre-treated using a PELCO easiGlow instrument (Ted Pella). The grid was flash frozen into liquid ethane using a Leica EM GP2 plunge freezer (Leica), with sensor back blotting for 5 seconds, under an environment of relative humidity of 95% and 6 °C.

### Cryo-EM data acquisition.

Grid screening was performed on a Talos Glacios microscope (Thermo Fisher Scientific). The best grid was used for a 24-hour data collection on a Titan Krios microscope (Thermo Fisher Scientific) with the post-column BioContinuum energy filter (Gatan) and a K3 direct electron detector (Gatan). A total of 6,817 movies were collected using SerialEM at a nominal magnification of 105,000x and pixel size 0.827 Å, with a 10 eV imaging filter slit.^72^ The accumulated total dose was ~50 e^−^/Å^2^ for each movie stack and fractionated into 50 frames. The defocus range of the images was set to be −0.8 to −2.2 μm.

### Cryo-EM data processing.

Cryo-EM data were processed in Relion.^73-75^ Raw movies were motion-corrected and dose-weighted, then averaged into single micrographs. The Contrast Transfer Function (CTF) parameters were calculated using CTFFIND4.^76^ 3,348,297 particles were auto-picked by Topaz, using a model trained with the particles manually picked from 20 micrographs.^77^ After two rounds of 2D classification, 990,497 particles remained. The initial 3D model was generated using selected 2D classes, assuming it is a left-handed helix with a crossover of 330 nm. After multiple rounds of 3D classification, particle polishing, and CTF refinement, 3D auto-refine with a stack of 37,444 particles and post-processing generated a reconstruction with an overall resolution of 2.3 Å, according to the gold-standard Fourier shell correlation (FSC) using the 0.143 criterion.^78^ The fibril structure adopts a C2 symmetry, with a refined helical twist of −2.97 degrees and a helical rise of 4.76 Å. The final map used for model building and refinement was sharpened with DeepEMhancer.^79^ More details are listed in the cryo-EM Table S2.

### Preparation of sample for ^111m^Cd PAC spectroscopy

^111m^Cd was produced by irradiation of ^108^Pd with α-particles at the University Hospital cyclotron in Copenhagen and separated from the target as described previously.^80^ This led to a solution of ca. 100 MBq ^111m^Cd in ca. 50 μL MilliQ water at the start of the chemical sample preparation. The sample for PAC spectroscopy was prepared by mixing 20 μL of the aqueous solution of ^111m^Cd with 5 μL 5 mM CdCl_2_ and 20 μL 1 M HEPES, pH 8. Next, MilliQ water was added to get a sample volume of 100 μL, and 100 μL 4 mM Ac-QFHFHWG-NH_2_ fibrils in suspension in 10 mM HCl solution, to achieve final concentrations of 0.1 mM CdCl_2_, 2 mM QFHFHWG (fibrils), 100 mM Hepes in a total volume of 200 μL. pH was measured after the PAC experiment to be 7.7. The sample was left to incubate for 15 min before the PAC measurement, and the sample was at room temperature during the measurement.

### PAC instruments and data analysis

The general theory of PAC spectroscopy may be found in the literature.^81,82^ An analogue PAC setup with six BaF_2_ detectors was used.^83^ The time resolution was 0.9 ns and the time-per-channel was 0.562 ns. The distance from the sample center was 26.9 mm to the detector heads. Data analysis was carried out with the Winfit and Prelude programs (provided by Prof. T. Butz) using 550 data points (excluding the first 5 points due to systematic errors in these). A Lorentzian line shape accounted for line broadening, given by the parameter *δ*, due to a static distribution of EFGs. Fourier transformation of the data and fits were carried out using 550 points and a Keiser-Bessel function with the window parameter equal to 4. The angular overlap model adapted to analysis of PAC data was applied to predict the PAC parameters given the experimentally determined structure of the Ac-QFHFHWG-NH_2_ fibrils.^84^

## Supplementary Material

This is a list of supplementary files associated with this preprint. Click to download.

" ConservedcatalyticmotifsencodeenzymescombinedSIv3.pdf

**Supplementary information** The online version contains supplementary material available at https://doi.org/

## Figures and Tables

**Fig. 1 ∣:**
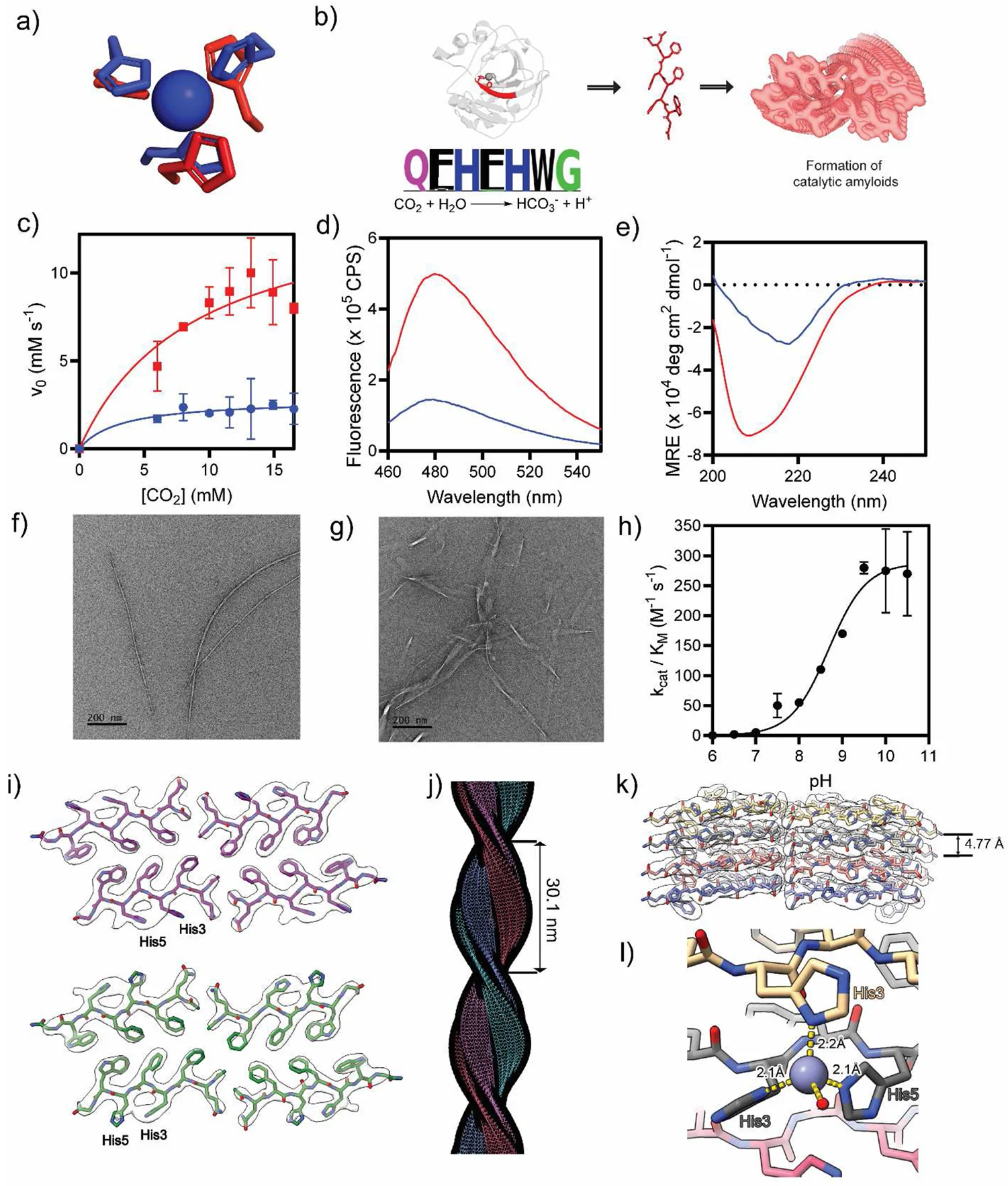
Carbonic anhydrase-like activity of amyloid designs a) Overlaid zinc binding site of carbonic anhydrase (PDB 1CA2) and Ac-IHVHLQI-NH_2_ (PDB 5UGK). b) An amyloid-like sequence can be identified within the active site of carbonic anhydrase, which self-assembles to form catalytic amyloids. c) Kinetic plots showing CO_2_ hydration activity of the Ac-QFHFHWG-NH_2_ fragment (blue) and the evolved Ac-FYFHFHFQF-NH_2_ (red). 2.5 μM peptide, 2.5 μM Zn^2+^, 50 mM CHES, pH 9.5 d) Thioflavin T fluorescence assay of the peptides Ac-QFHFHWG-NH_2_ (blue) Ac-FYFHFHFQF-NH_2_ (red) e) Circular dichroism of the peptides Ac-QFHFHWG-NH_2_ (blue) Ac-FYFHFHFQF-NH_2_ (red) 100 μM peptide, 5 mM HEPES, pH 8 .100 μM peptide, 25 μM ThT, 25 mM Tris, pH 8. Transmission electron micrographs of f) Ac-QFHFHWG-NH_2_ or g) Ac-FYFHFHFQF-NH_2_ (100 μM peptide, 25 mM Tris, pH 8). h) pNPA hydrolysis efficiency of Ac-FYFHFHFQF-NH_2_ as a function of pH. 25 μM Ac-FYFHFHFQF-NH_2_, 25 mM buffer. i) Single layer of the Ac-QFHFHWG-NH_2_ fibril model fitted into the cryo-EM density maps of conformation 1 (purple) and conformation 2 (green). His3 and His5, which adopt alternative conformations, are indicated. j) A synthetic four-start fibril reconstructed from the cryo-EM structure model. K) Side view of a four-layer fibril model fitted into the cryo-EM density map. l) Putative Zn^2+^ coordination site located between two layers, adopting a tetrahedral geometry. Zn is shown as a gray sphere, and water as a small red sphere.

**Fig. 2 ∣:**
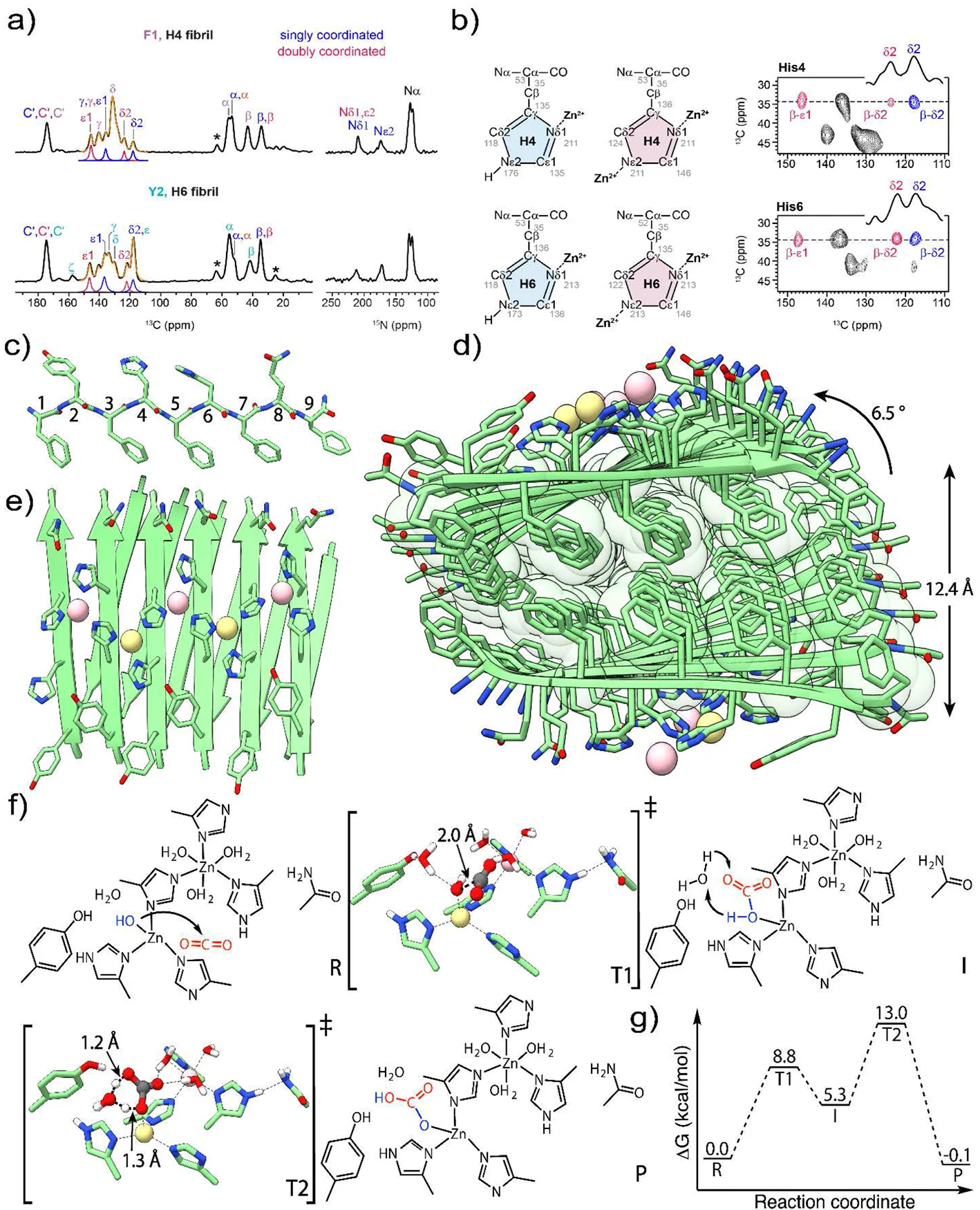
Zinc coordination by His4 and His6 residues in Ac-FYFHFHFQF-NH2 fibrils. a) 1D ^13^C and ^15^N CP spectra of two catalytic fibrils at pH8 with different labeling schemes: Phe1 and His4 (top) as well as Tyr2 and His6 (bottom). Spinning sidebands are indicated with asterisks. Spectral deconvolution was conducted for of ^13^C CP spectra to better resolve the signals of singly (blue) and doubly (magenta) coordinated His residues. The simulated spectra (yellow) closely matched the experimental data (black). Carbon sites from other two residues, Phe1 and Tyr2 are marked in purple and turquoise, respectively. b) Summary of zinc-coordination and ^13^C/^15^N chemical shifts of His4 and His6. C) Selected regions of 2D ^13^C-^13^C correlation spectra highlighting the Cβ-Cδ2 (β-δ2) cross peaks for singly (blue) and doubly (magenta) coordinated His4 (top) and His6 (bottom) residues. Dashed lines show the positions where 1D cross sections were extracted, showing almost equal peak area for the Cδ2 sites in singly and doubly coordination. d,e) MD equilibrated structure of **6**-Zn^2+^ model. f) QM/MM optimized structures of all maxima and minima for the reaction. g) Potential Energy Surface diagram of the reaction including energies in kcal/mol.

**Fig. 3 ∣:**
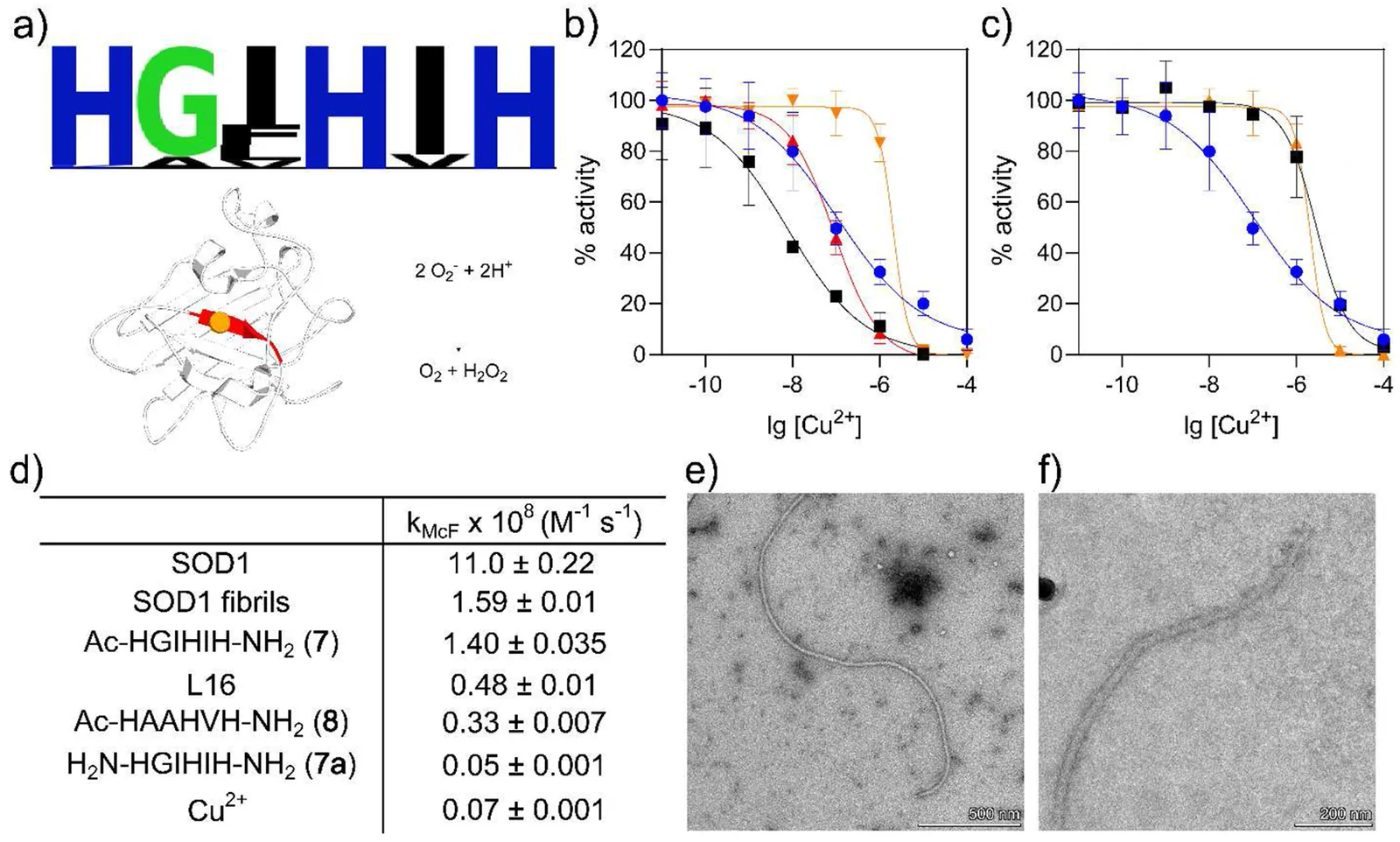
Superoxide dismutase activity of amyloid species. A- structure of SOD1 showing a conserved β-sheet at the metal binding site with a corresponding sequence alignment. B- McCord-Fridovich superoxide dismutase assay for bovine SOD1 (black), bovine SOD1 amyloids (red), Cu/Ac-HGIHIH-NH_2_ (7) (blue) and free Cu^2+^ (orange). Conditions: 50 mM HEPES, pH 7.8, 25 μM xanthine, 0.1 μM xanthine oxidase, 250 μM NBT. C- McCord-Fridovich superoxide dismutase assay for 7 (blue) and 7a (black), and free copper in buffer (orange). Conditions: 50 mM HEPES, pH 7.8, 25 μM xanthine, 0.1 μM xanthine oxidase, 250 μM NBT. D- Experimentally determined kMcF values. TEM micrographs of E- Fibrillated bovine superoxide dismutase (30 μM protein, 50 mM HEPES, pH 7.8) and F- Ac-HGIHIH-NH_2_ (100 μM fibrils, 100 μM Cu^2+^+, 50 mM HEPES, pH 7.8).

**Table 1 ∣:** Kinetic parameters for pNPA hydrolysis (pH 8) and CO_2_ hydration (pH 9.5). Conditions: 50 mM CHES, pH 9.5, 25 μM thymol blue.

	pNPA hydrolysis			CO_2_ hydration				
	k_cat_ x 10^−2^ (s^−1^)	K_M_ (mM)	k_cat_/K_M_ (M^−1^ s^−1^)	k_cat_ (s^−1^)	K_M_ (mM)	k_cat_/K_M_ x 10^5^ (M^−1^ s^−1^)	Specific activity (kat g^−1^)^a^	Ref.
Ac-QFHFHWG-NH_2_ **(1)**	0.3 ± 0.025	0.3 ± 0.07	9.2 ± 2.6	3,170 ± 960	9.5 ± 4.5	3.3 ± 1.2	1.6	This work
Ac-FQFHFHF-NH_2_ **(2)**	0.4 ± 0.03	0.5 ± 0.07	8.4 ± 1.3	8,550 ± 3110	23 ± 8.9	3.7 ± 0.7	3.7	This work
Ac-FHFHFQF-NH_2_ **(3)**	1.1 ± 0.06	1.1 ± 0.1	9.5 ± 1.0	5,230 ± 1190	8.2 ± 3.1	6.4 ± 2.0	3.1	This work
Ac-FYFHFHF-NH_2_ **(4)**	0.3 ± 0.03	0.15 ± 0.06	18.7 ± 7.8	3190 ± 1390	7.8 ± 6	4.1 ± 2.5	1.7	This work
Ac-FHFHFYF-NH_2_ **(5)**	0.9 ± 0.16	0.3 ± 0.14	29.6 ± 14.8	1270 ± 400	7.2 ± 4	1.8 ± 0.77	1.8	This work
Ac-FYFHFHFQF-NH_2_ **(6)**	1.2 ± 0.12	0.13 ± 0.06	92 ± 43	11,760 ± 2876	9.1 ± 3.6	12.9 ± 3.9	4.3	This work
Phe-Zn-Phe^b^	0.2	0.17	77	7.8	8.1	0.01	0.013	*8*
Hg/Zn TriL9CL23H	n.r.	n.r.	1.4	1,800 ± 430	10.0 ± 2.4	1.8 ± 0.3	0.095	*17*
Zn^II^α_3_DH_3_	n.r..	n.d.	n.r.	134 ± 8	3.5 ± 0.6	0.4 ± 0.05	0.011	*18*
HCA I^c^	22 ± 1	3 ± 0.01	753 ± 31	200,000	4.0	500	5.6	*16,24*
HCA II^d^	81 ± 6.1	30.5 ± 2.1	2607 ± 85	1,400,000	9.3	1500	31	*16,24*
HCA III	n.r.	n.r.	n.r.	10,000	33.0	3	.11	*16*

a Specific activity values calculated from the kinetic parameters at 16 mM of CO_2_.

b 20 mM Tris, pH 8, 50 μM phenol red

c pH 7.5

d pH 7.4 n.r. – not reported

## Data Availability

The cryo-EM density maps have been deposited in the Electron Microscopy Data Bank (EMDB) under accession code xxx (submission in progress). The coordinates generated in this study are deposited in the Protein Data Bank (PDB) under accession code xxxx (submission in progress). Biological materials are available on request. All data supporting the findings of this study are available from the Zenodo repository at 10.5281/zenodo.21986196. The repository contains the datasets underlying the figures and conclusions of this study.

## Code availability

Code used for sequence analysis and data processing is available at GitHub https://github.com/liamrmarshall7-pixel/SequenceConservation.git.
